# Retraction: Agarwal *et al.* Dynamic Action of Carotenoids in Cardioprotection and Maintenance of Cardiac Health, *Molecules* 2012, *17*, 4755-4769

**DOI:** 10.3390/molecules19033850

**Published:** 2014-03-25

**Authors:** Derek McPhee

**Affiliations:** Editor-in-Chief of *Molecules*, Multidisciplinary Digital Publishing Institute (MDPI), Klybeckstrasse 64, Basel CH-4057, Switzerland; E-Mail: mcphee@mdpi.com

We were recently alerted by an anonymous tip that at least two paragraphs in the title review [1] appeared to be copied verbatim from papers by other authors without attribution. Upon checking these facts we have established that in addition to the parts brought to our attention, large portions of the review are made up of paragraphs copied verbatim from other reviews or books, sometimes cited, but mostly presented without any references other than to the corresponding original primary literature. While some reproduction of properly quoted previous material might be expected in a review article like [1], wholesale plagiarism obviously does not meet that standard and consequently this review is now being retracted and marked accordingly for the scientific record. The corresponding author Dipak K. Das with whom ultimate responsibility for the contents of the paper resided passed away in September 2013. As a COPE member, MPDI takes very seriously the responsibility to ensure the addition of high quality original scientific works to the field of scholarly publication. We apologize to our readership and the authors and publishers of the plagiarized material for the fact that this went undetected until now. We would also like to thank the reader who brought this to our attention, and encourage others with similar suspicions to contact us directly and promptly so we may investigate and respond to these issues in a timely fashion.
